# Characterization of Synthetic Tf2 as a Na_V_1.3 Selective Pharmacological Probe

**DOI:** 10.3390/biomedicines8060155

**Published:** 2020-06-11

**Authors:** Mathilde R. Israel, Thomas S. Dash, Stefanie N. Bothe, Samuel D. Robinson, Jennifer R. Deuis, David J. Craik, Angelika Lampert, Irina Vetter, Thomas Durek

**Affiliations:** 1 Institute for Molecular Bioscience, The University of Queensland, St. Lucia, QLD 4072, Australia; mathilde.israel@uqconnect.edu.au (M.R.I.); thomasdash123@gmail.com (T.S.D.); s.robinson@imb.uq.edu.au (S.D.R.); j.deuis@uq.edu.au (J.R.D.); d.craik@imb.uq.edu.au (D.J.C.); 2 Institute of Physiology, Medical Faculty, RWTH Aachen University, 52074 Aachen, Germany; stbothe@ukaachen.de (S.N.B.); alampert@ukaachen.de (A.L.); 3 Research Training Group 2416 MultiSenses-MultiScales, RWTH Aachen University, 52074 Aachen, Germany; 4 Research Training Group 2415 ME3T, RWTH Aachen University, 52074 Aachen, Germany; 5 School of Pharmacy, The University of Queensland, Woolloongabba, QLD 4102, Australia

**Keywords:** Tf2, sodium channel, Na_V_1.3, Na_V_1.9, scorpion, toxin

## Abstract

Na_V_1.3 is a subtype of the voltage-gated sodium channel family. It has been implicated in the pathogenesis of neuropathic pain, although the contribution of this channel to neuronal excitability is not well understood. Tf2, a β-scorpion toxin previously identified from the venom of *Tityus fasciolatus*, has been reported to selectively activate Na_V_1.3. Here, we describe the activity of synthetic Tf2 and assess its suitability as a pharmacological probe for Na_V_1.3. As described for the native toxin, synthetic Tf2 (1 µM) caused early channel opening, decreased the peak current, and shifted the voltage dependence of Na_V_1.3 activation in the hyperpolarizing direction by −11.3 mV, with no activity at Na_V_1.1, Na_V_1.2, and Na_V_1.4-Na_V_1.8. Additional activity was found at Na_V_1.9, tested using the hNav1.9_C4 chimera, where Tf2 (1 µM) shifted the voltage dependence of activation by −6.3 mV. In an attempt to convert Tf2 into an Na_V_1.3 inhibitor, we synthetized the analogue Tf2[S14R], a mutation previously described to remove the excitatory activity of related β-scorpion toxins. Indeed, Tf2[S14R](10 µM) had reduced excitatory activity at Na_V_1.3, although it still caused a small −5.8 mV shift in the voltage dependence of activation. Intraplantar injection of Tf2 (1 µM) in mice caused spontaneous flinching and swelling, which was not reduced by the Na_V_1.1/1.3 inhibitor ICA-121431 nor in Na_V_1.9^-/-^ mice, suggesting off-target activity. In addition, despite a loss of excitatory activity, intraplantar injection of Tf2[S14R](10 µM) still caused swelling, providing strong evidence that Tf2 has additional off-target activity at one or more non-neuronal targets. Therefore, due to activity at Na_V_1.9 and other yet to be identified target(s), the use of Tf2 as a selective pharmacological probe may be limited.

## 1. Introduction

Voltage-gated sodium channels (VGSCs) are large pore-forming transmembrane-spanning proteins with four homologous domains (I-IV) that regulate the influx of Na^+^ ions across neuronal membranes in response to local changes in voltage [1]. This Na^+^ influx is essential for action potential initiation and propagation in electrically excitable cells and crucial for the physiological function of neurons. In humans, nine different isoforms with relatively high sequence homology have been identified (Na_V_1.1–Na_V_1.9), each with discrete expression profiles and distinctive biophysical properties [2]. While several VGSC isoforms expressed in peripheral sensory neurons, such as Na_V_1.7, Na_V_1.8, and Na_V_1.9, have a well-established role in somatosensation and nociception, the contribution of Na_V_1.3 to peripheral neuronal excitability is less well understood.

Na_V_1.3 is a tetrodotoxin (TTX)-sensitive channel with fast-activating and fast-inactivating currents capable of rapid repriming and sustaining repetitive firing [3]. Interestingly, Na_V_1.3 is expressed at very low levels in adult rodent dorsal root ganglion (DRG) neurons but is upregulated in DRGs following nerve injury or streptozotocin-induced diabetes, suggesting the channel may contribute to the development and maintenance of neuropathic pain [4,5,6]. Indeed, shRNA-mediated knockdown of Na_V_1.3 in DRG neurons attenuates the development of mechanical allodynia in rodent models of spared nerve injury (SNI) and diabetic neuropathy [7,8], while global Na_V_1.3 knockout has confounding results, with minimal effect on the development of mechanical allodynia following chronic constriction injury (CCI), spinal nerve transection (SNT), and spinal nerve ligation (SNL) [9,10]. Therefore, selective pharmacological modulators of Na_V_1.3 are required to further elucidate the contribution of the channel to neuronal excitability in physiological and pathological pain states.

Venoms from scorpions, spiders, and cone snails are a rich source of novel bioactive peptides with activity at ion channels. Many venom-derived peptides have high potency and selectivity for VGSCs and have provided us with unique insights into VGSC gating, structure, and function [11]. Scorpion peptides that act on VGSCs can be functionally distinguished as α- or β-scorpion toxins, depending on how they modulate channel opening or closing. α-Scorpion toxins cause a marked delay in fast inactivation, resulting in prolonged Na^+^ influx; whereas, β-scorpion toxins shift the voltage dependence of activation to more hyperpolarized potentials, resulting in toxin-bound channels that open closer to the resting membrane potential [12,13]. Tf2 is a β-scorpion toxin originally identified from the venom of *Tityus fasciolatus* that, at a concentration of 1 µM, selectively activates Na_V_1.3, with no effect at the other Na_V_ subtypes (Na_V_1.1, Na_V_1.2, and Na_V_1.4–Na_V_1.8 tested) expressed in *Xenopus* oocytes [14]. We therefore hypothesized that Tf2 could be used as a pharmacological tool to assess the contribution of Na_V_1.3 to the excitability of peripheral sensory neurons. The remaining sodium channel subtype Nav1.9 is historically hard to express, but recent progress showed that the chimera of the human Nav1.9 with the C-terminus of Nav1.4 (hNav1.9_C4) reveals reasonable currents in HEK293 cells [8]. As Tf2 is not thought to interact with the C-terminus, this chimera is a valuable tool for testing its influences on Nav1.9 gating.

Here, we report the chemical synthesis of Tf2 and the mutant Tf2[S14R], along with previously undescribed β-scorpion activity of Tf2 at hNa_V_1.9_C4 in vitro. We show for the first time that Tf2 causes Ca^2+^ influx in small, medium, and large diameter mammalian sensory neurons and explore the activity of Tf2 on the peripheral somatosensory system in vivo.

## 2. Experimental Section

### 2.1. Peptide Synthesis

Tf2 was synthesized in two segments consisting of Tf2(1–26)-α-thioester and Tf2(27–62), which were linked by native chemical ligation. Tf2(1–26)-α-thioester was synthesized via Boc chemistry on trityl-associated mercaptopropionic acid lysine resin to yield the C-terminal α-thioester upon hydrogen fluoride (HF, BOC Australia, Sydney, NSW, Australia) cleavage. Tf2(27–62) was synthesized using Fmoc chemistry on Rink amide resin to yield the C-terminally amidated peptide segment after trifluoroacetic acid (TFA, Auspep, Melbourne, VIC, Australia) cleavage. The cleaved crude peptides were precipitated and washed twice with cold diethyl ether. The peptides were then dissolved in 50% acetonitrile, 0.1% TFA (*v*/*v*) in water and lyophilized. Peptides were purified by preparative reverse-phase (RP) HPLC on a Shimadzu Prominence system (Sydney, NSW, Australia). HPLC fractions containing the desired peptide (judged by HPLC/MS) of similar purity were pooled, lyophilized, and stored at −20 °C.

Tf2(1–26)-α-thioester: KEGYAMDHEGCKFSCFIRPSGFCDGY-[COS-CH2-CH2-CO]-Lys, expected MW (assuming average isotope composition): 3164.5 Da, observed MW: 3164.4 ± 0.5 Da. Tf2(27–62): CKTHLKASSGYCAWPACYCYGVPSNIKVWDYATNKC-NH_2_, expected MW (assuming average isotope composition): 4031.7 Da, observed MW: 4031.2 ± 0.5 Da.

Native chemical ligation was done as previously described [11]. Briefly, 8.7 μmol (35.3 mg) of peptide Tf2(27–62) and 8.7 μmol (27.6 mg) of Tf2(1–26)-α-thioester were dissolved in 8.7 mL of ligation buffer consisting of 6 M guanidine hydrochloride, 0.2 M Na_2_HPO_4_, 40 mM Tris(2-carboxyethyl)phosphine hydrochloride (TCEP), and 50 mM 4-mercaptophenylacetic acid (MPAA), pH 7.25. The solution was stirred under nitrogen for 16 h and the reaction progress was monitored by HPLC-UV. The reduced full-length Tf2 (1–62) ligation product was isolated via RP-HPLC on an Agilent Zorbax 300SB, C3 column (9.4 × 250 mm, 5 μm Phenomenex Australia, Sydney, NSW, Australia). Yield: 42.5 mg; expected MW (assuming average isotope composition): 6963.0 Da, observed MW: 6962.8 ± 1.0 Da.

For oxidative folding of Tf2, 1.7 μmol (15 mg) of reduced Tf2 were dissolved in 3.75 mL of 6 M guanidine hydrochloride. Folding was initiated by diluting the peptide solution with 150 mL of folding buffer (0.1 M Tris, 0.5 M arginine, 1 M urea, 10 mM reduced glutathione, 1 mM oxidized glutathione, pH 8.0) and stirring for 72 h at 4 °C. Oxidized Tf2 was then purified by RP-HPLC on a Agilent Zorbax 300SB, C3 column (9.4 × 250 mm, 5 μm, Phenomenex Australia, Sydney, NSW, Australia). Yield: 2.2 mg. Calculated MW (assuming most abundant isotope composition): 6949.03 Da, observed MW: 6949.12 ± 0.20 Da. All reagents were obtained from Sigma-Aldrich (St Louis, MI, USA) unless otherwise stated.

### 2.2. Cell Culture

Human embryonic kidney (HEK) 293 cells that constitutively express the human VGSC channel α subunits Na_V_1.1–1.7/β1 (SB Drug Discovery, Glasgow, UK) were maintained in minimum essential media (MEM) supplemented with 10% fetal bovine serum (FBS) and 2 mM l-glutamine, along with the selection antibiotics blasticidin, geneticin, and zeocin as recommended by the manufacturer. Chinese hamster ovary (CHO) cells expressing hNa_V_1.8/β3 via a tetracycline inducible system (ChanTest, Cleveland, OH, USA) were maintained in MEM, 10% FBS, and 2 mM l-glutamine. Tetracycline (1 µg mL^−1^) was added to culture media 48–72 h prior to assay to induce hNa_V_1.8 expression. Cells were passaged every 3–4 days after reaching 70–80% confluence with TrypLE Express (Thermo Fisher Scientific, Scoresby, VIC, Australia) and grown in an incubator at 37 °C with 5% CO_2_.

ND7/23 cells (Sigma-Aldrich, St. Louis, MO, USA) were maintained in high glucose Dulbecco’s Modified Eagle Medium (DMEM; Gibco, Thermo Fisher Scientific, Waltham, MA, USA) with a glucose level of 4.5 g/L and supplemented with 10% FBS (Biochrom AG, Berlin, Germany). ND7/23 cells were transiently transfected with 2.75 µg hNa_V_1.9_C4 plasmid (chimera of human Na_V_1.9 and the C-terminus of rat Na_V_1.4; pCDNA3 vector [8]), 0.25 μg of green fluorescent protein (GFP) DNA (Lonza, Basel, Switzerland), and 6 μL of JetPEI reagent (Polyplus Transfection, Illkirch, France). GFP was co-transfected to detect transfected cells via green fluorescence. Then, 24 h after transfection, cells were split either onto glass coverslips coated with poly-D-lysine (PDL) hydrobromide (Sigma-Aldrich) for treatment with 1 µM Tf2, or split into plastic petri dishes for control conditions. After recovering for 2–3 h, the cells were recorded with whole-cell patch clamp. Cells were passaged every 3–4 days after reaching 70–80% confluence with TrypLE Express (Thermo Fisher Scientific, Scoresby, VIC, Australia) in the case of HEK and CHO cells, and with accutase (Sigma-Aldrich) in the case of the ND7/23 cells. All cells were grown in an incubator at 37 °C with 5% CO_2_.

### 2.3. Fluorescent Membrane Potential Assay

To rapidly assess the activity of Tf2 and Tf2[S14R] at hNa_V_1.1–1.7, we utilized the FLIPR^Tetra^ fluorescent imaging plate reader (Molecular Devices, Sunnyvale, CA, USA). Cells were plated in 384-well black-walled plates at a density of 10,000–15,000 per well in normal growth media 24–48 h before membrane potential experiments. Cells were loaded with red membrane potential dye (Molecular Devices) diluted in physiological salt solution (PSS; 140 mM NaCl, 11.5 mM glucose, 5.9 mM KCl, 1.4 mM MgCl_2_, 1.2 mM NaH_2_PO_4_, 5 mM NaHCO_3_, 1.8 mM CaCl_2_, 10 mM HEPES) and incubated for 30 min at 37 °C as per the manufacturer’s instructions. All synthetic toxins were diluted in PSS and 0.1% bovine serum albumin (BSA) to avoid adsorption to plastic surfaces. Changes in membrane potential (excitation/emission 510–545 nm/565–625 nm) were recorded each second for 5 min after the addition of toxin and the area under the curve (AUC) was computed using ScreenWorks (Molecular Devices, Version 3.2.0.14).

### 2.4. Whole Cell Patch-Clamp Electrophysiology

Whole cell patch-clamp experiments on hNa_V_1.3, hNa_V_1.7, and hNa_V_1.8 cell lines were performed using the QPatch-16 automated electrophysiology platform (Sophion, Ballerup, Denmark). This set-up utilizes 16-well planar chips plates (QPlates, Sophion) with a standard resistance of 2 ± 0.02 mΩ. Cells were expanded in T-175 flasks and maintained in 37 °C and 5% CO_2_ 48–72 h prior to assay. On the day of experiment, cells were harvested with TrypLE Express and resuspended in DMEM with 25 mM HEPES, 100 U/mL penicillin-streptomycin, and 0.04 mg/mL trypsin inhibitor from *Glycine max* (soybean) and stirred for 30–60 min prior to use.

The external solution (ECS) for all automated VGSC recordings contained (in mM): NaCl (140), KCl (4), CaCl_2_ (2), MgCl_2_ (1), HEPES (10), TEA-Cl (20), and glucose (10) containing 0.1% BSA. The pH was adjusted to 7.4 with NaOH and osmolarity adjusted to 305 mOsm with sucrose. The intracellular solution (ICS) consisted of (in mM): CsF (140), EGTA/CsOH (1/5), HEPES (10), and NaCl (10) adjusted to pH 7.4 with CsOH and 320 mOsm with sucrose. The cell positioning pressure was set to −60 mbar, minimum seal resistance 0.1 GΩ, holding pressure −20 mbar, and currents were filtered at 25 kHz (8th order Bessel, cut off 5 kHz). A standard P/4 leak subtraction protocol was included, and as such, leak subtracted and non-leak subtracted currents were acquired in parallel.

After obtaining the stable whole-cell configuration, voltage clamp experiments proceeded as follows from a holding potential of −90 mV. All protocols included a brief (15 ms) pre-conditioning pulse to 0 mV followed by a 120 ms recovery to allow voltage-sensor trapping by β-scorpion toxins [15]. Current–voltage (IV) relationships were determined using a series of 500 ms pulses ranging from −90 to +55 mV (in 5 mV steps), followed by a 20 ms pulse to −20 mV for hNa_V_1.3 and hNa_V_1.7 or +10 mV for hNa_V_1.8 to assess the voltage dependence of fast inactivation. Peak current values were normalized to a buffer control. Toxins were diluted in ECS with 0.1% BSA and incubated for 5 min prior to recording.

Whole-cell patch-clamp experiments on ND7/23 cells transfected with hNav1.9_C4 were performed at room temperature, using an EPC-10 USB amplifier and PatchMaster software (HEKA Elektronik, Lambrecht, Germany) and analysis was done using Igor Pro 6.3 (WaveMetrics, Lake Oswego, OR, USA). Glass pipettes were prepared with a DMZ puller (Zeitz Instruments, Martinsried, Germany) to a resistance of 0.9 to 2.5 MΩ and filled with ICS. The ICS for hNav1.9_C4 recordings consisted of (in mM): CsF (140), EGTA (1), HEPES (10) and NaCl (10), sucrose (18) adjusted to pH 7.33 with CsOH, and 310 mOsm with additional sucrose. The ECS for hNav1.9_C4 recordings contained (in mM): NaCl (140), KCl (3), CaCl_2_ (1), MgCl_2_ (1), HEPES (10), glucose (20), and 0.1 % BSA. The pH was adjusted to 7.4 with NaOH and osmolarity adjusted to 305 mOsm with glucose. TTX (500 nM) was added to the ECS to block endogenous Na^+^ currents in ND7/23 cells. Tf2 (1 µM) was diluted in ECS with 0.1% BSA and incubated for 5 min prior to recording. Capacitive transients were cancelled and series resistance (≤6 MΩ) was compensated by at least 70%. Leak current was subtracted online using a P/4 procedure following each test pulse. Signals were sampled with 100 kHz and filtered at 10 kHz for activation and fast inactivation protocols and at 30 kHz for the deactivation protocol. Recording protocols were started 5 min after establishing the whole-cell configuration to allow for current stabilization. The holding potential for all recordings was −120 mV. The voltage dependence of activation was measured using a series of 40 ms pulses ranging from −120 mV to 30 mV in 10 mV steps at an interval of 5 s. The voltage dependence of steady-state fast inactivation was measured using a series of 500 ms pre-pulses ranging from −160 to −20 mV, in steps of 10 mV, followed by a 40 ms depolarizing test pulse at −40 mV. The voltage dependence of deactivation was measured using a series of 1.5 ms depolarizing pre-pulses to −20 mV. This was followed by a repolarization test pulse ranging from −130 to −20 mV, in 10 mV steps, at an interval of 5 s.

### 2.5. Ca^2+^ Imaging of Isolated Dorsal Root Ganglion Neurons

Primary cultures of mouse DRG neurons were prepared as previously described [16,17]. Briefly, DRG ganglia, removed from all spinal levels of 4-week-old male C57BL/6 mice, were incubated in DMEM (Gibco, Waltham, MD, USA) with 1 mg mL^−1^ Collagenase IV (Gibco, Life Technologies, NY, USA) for 90 min at 37 °C, then triturated with fire-polished Pasteur pipettes and plated in 96-well plates coated with PDL (Corning, ME, USA). After 1 h, wells were flooded with DMEM supplemented with 10% FBS and pencillin/streptomycin (Gibco, Waltham, MD, USA) and maintained for 16–24 h before assay. Cells were loaded with Fluo-4 AM calcium indicator, according to the manufacturer’s instructions (ThermoFisher Scientific, MA, USA). After loading (1 h), the dye-containing solution was replaced with assay solution (1 x Hanks’ balanced salt solution, 20 mM HEPES). Fluorescence corresponding to [Ca^2+^]*_i_* of 100–200 DRG cells per experiment was monitored in parallel using a Nikon Ti-E Deconvolution inverted microscope (Nikon, Tokyo, Japan), equipped with a Lumencor Spectra LED Lightsource (Lumencor, Beavertown, OR, USA). Images were acquired at 10x objective at 1 frame/s (excitation 485 nm, emission 521 nm). For each experiment, baseline fluorescence was monitored for 30 s. At 30 s, 60 s, and 180 s, assay solution was replaced with fresh assay solution (as a negative control), Tf2 (1 µM in assay solution), and KCl (30 mM in assay solution), respectively. Experiments with TTX and Tf2[S14R] were performed in the same way. Cells responding to KCl and/or Tf2 were considered neuronal and grouped according to size: Large (>600 µm^2^), medium (300–600 µm^2^), and small diameter (<300 µm^2^). Experiments involving the use of mouse tissue were approved by The University of Queensland Animal Ethics Committee. Data were derived from 2–3 independent experiments, and plotted using GraphPad Prism 8. Where differences were tested, unpaired *t*-tests were used.

### 2.6. Animals

For behavioral assessment, we used adult male wildtype or homozygous Na_V_1.9-deficient (Na_V_1.9^-/-^) mice aged 6–8 weeks (~25g) on a C57BL/6J background. Homozygous Nav1.9 knockout mice on a C57BL/6J background were generated using CRISPR/Cas9 technology by the Queensland Facility for Advanced Genome Editing (The University of Queensland, Australia). In brief, two sgRNAs were designed to target exon 2 of the *Scn11a* gene. The deletion of exon 2 generated a frame shift mutation resulting in the introduction of stop codons in exon 3. The guide sgRNAs were microinjected together with Cas9 mRNA into fertilized eggs collected from C57BL/6J mice and transferred into pseudopregnant surrogate CD1 female mice. Offspring born to the foster mothers were genotyped by PCR (see below) and DNA sequencing. One F0 male mouse was bred to C57BL/6J female mice to generate F1 heterozygous mice, and F1 heterozygous mice were inbred to obtain homozygous mice. Genomic DNA was extracted from ear clippings and mice were genotyped by PCR using the following primers (forward: 5′-GCTGCTCAGACACTCACAGT-3′) and (reverse: 5′ATTCTGCCACCAGAGACTGC-3′) by the AEGRC genotyping and sequencing facility (The University of Queensland). Animals were housed in groups of 3 or 4 per cage, under 12-h light-dark cycles, and had standard rodent chow and water ad libitum. Age-matched controls were used for studies involving knockout animals. All behavioral assessment was performed by a blinded observer unaware of the genotype and/or treatments received.

Ethical approval for in vivo experiments was obtained from The University of Queensland Animal Ethics Committee prior to experimentation. All animals experiments were conducted in accordance with local and national regulations, including the International Association for the Study of Pain Guidelines for the Use of Animals in Research in agreement with the Animal Care and Protection Regulation Qld (2012), and the Australian Code of Practice for the Care and Use of Animals for Scientific Purposes, 8th edition (2013) (TRI/IMB/093/17, 31 March 2017; IMB/PACE/421/18, 18 October 2018).

### 2.7. Behavioral Assessment

First, Tf2 (1 µM), Tf2[S14R] (10 µM), or ICA-121431 (500 nM) were diluted in saline with 0.1% *w*/*v* BSA and administered either alone or in combination by intraplantar (i.pl) injection into the left hind paw in a volume of 40 µL under light isoflurane (3 %) anesthesia. Mice were then placed individually into polyvinyl boxes (10 × 10 × 10 cm) and recorded by video for 20 min post-injection. The number of spontaneous pain behaviors, including licks, shakes, and flinches, were counted later by a blinded observer. Once spontaneous pain behavior had ceased, mechanical thresholds were assessed using an electronic von Frey apparatus (MouseMet Electronic von Frey, TopCat Metrology, Ely, UK), heat thresholds were assessed using the thermal probe test (MouseMet Thermal), and weight bearing was assessed using the Catwalk XT (Noldus Information Technology, Wageningen, The Netherlands) as previously described [18,19]. To quantify weight bearing, the parameter ‘mean intensity of the 15 most intense pixels’ was used as a surrogate measure.

### 2.8. Paw Thickness

In a separate cohort of animals, paw thickness was measured along the distal–proximal axis at the metatarsal level using a digital vernier caliper (Kincrome, Vic, Australia) 10 min after i.pl. injection of Tf2 (1 µM) or Tf2[S14R] (10 µM).

### 2.9. Data Analysis

Data were plotted and analyzed by GraphPad Prism, versions 8.2.0 and 8.4.2. Statistical significance was defined as *p* < 0.05 and was determined by *t*-test or one-way ANOVA with Dunnett’s post-test, as indicated. In case of deactivation, multiple *t*-tests (one per row) were used without assuming equal standard deviation and with the Holm–Sidak method for correcting *p*-values for multiple comparisons. Concentration–response curves were fitted with a four-parameter Hill equation with a variable Hill coefficient. Conductance–voltage relationships for activation were calculated for each voltage step using $G=\frac{I}{\left(V-V_{rev}\right)}$ where V_rev_ is the reversal potential. Resulting data were fitted using a Boltzmann equation: G_Na_ =$\frac{G_{Na,max}}{1+e^{\left[\left(Vm-V\frac{1}{2}\right)\right]/k}}$; G_Na_ is the voltage-dependent sodium conductance, G_Na,max_ is the maximal sodium conductance, V_m_ is the membrane potential, V_1/2_ is the membrane potential at half-maximal activation, and k is the slope factor. Current–voltage relationships for inactivation were fitted using a Boltzmann equation: I_Na_ = $\frac{I_{Na,max}}{1+e^{\left[\left(Vm-V\frac{1}{2}\right)\right]/k}}$ I_Na_ is the voltage-dependent sodium current, I_Na,max_ is the maximal sodium current, V_m_ is the membrane potential, V_1/2_ is the half-maximal inactivation, and k is the slope factor. The time course of current decay at deactivating voltages over 10 ms was fitted by the double-exponential equation,$\;y=y_{0}+amp_{1}*e^{-\frac{x}{\tau_{1}}}+amp_{2}*e^{-\frac{x}{\tau_{2}}}$, where y_0_ is the current amplitude at steady state, amp_1_ and amp_2_ are the amplitude coefficient for the fast and slow time constants, and τ_1_ and τ_2_ are the fast and slow time constants. Data are expressed as the mean ± standard error of the mean (SEM).

## 3. Results

### 3.1. Chemical Synthesis of Tf2

We used chemical synthesis to prepare multi-milligram quantities of Tf2. This toxin is a relatively large (62 amino acid residues) and post-translationally modified protein, whose 3-D structure is stabilized by four disulfide crosslinks. The target polypeptide chain was split into two segments of manageable size (26 and 36 residues), which were assembled individually by solid-phase peptide synthesis and purified. The full-length polypeptide chain was obtained in good yield by native chemical ligation of the two segments. Folding of the 62 amino acid peptide chain and oxidation of the eight cysteines required extensive optimization and allowed the isolation of synthetic Tf2 in acceptable yields and excellent purity for further functional studies. Functional equivalence with venom-derived Tf2 was established through activity testing (see below) as well as partial assignment of NMR data, which are in agreement with the expected cysteine-stabilized αβ fold that is characteristic for this class of scorpion toxins (Figure S1).

### 3.2. Synthetic β-Scorpion Tf2 Causes Early Channel Opening at Na_V_1.3 and Na_V_1.9

**Figure 1:**
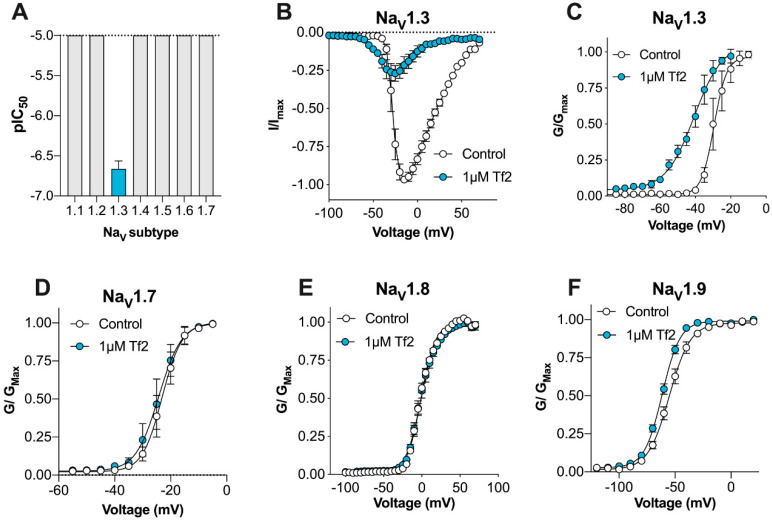
Synthetic Tf2 shifts the voltage dependence of activation at hNa_V_1.3 and hNa_V_1.9_C4. (**A**) Synthetic Tf2 retains selectivity for hNa_V_1.3, with no effect on hNa_V_1.1, hNa_V_1.2, or hNa_V_1.4–1.7 up to 10 µM in the FLIPR^Tetra^ membrane potential assay (*n* = 4–6 wells) (**B**) hNa_V_1.3 current–voltage relationship before (white circles) and after the addition of 1 μM Tf2 (blue circles). Tf2 causes early channel opening and an overall decrease in the peak current (*n* = 5 cells). (**C**) hNa_V_1.3 conductance–voltage relationship before (white circles) and after the addition of 1 μM Tf2 (blue circles). Tf2 causes a significant (∆ −11.3 mV) hyperpolarizing shift in the voltage dependence of activation and increases the slope factor (*n* = 4 cells). (**D**) hNa_V_1.7 conductance–voltage relationship before (white circles) and after the addition of 1 μM Tf2 (blue circles) (*n* = 4 cells). (**E**) hNa_V_1.8 conductance–voltage relationship before (white circles) and after addition of 1 μM Tf2 (blue circles) (*n* = 12 cells). (**F**) hNav1.9_C4 conductance–voltage relationship incubated with vehicle (0.1% BSA, white circles) or with 1 μM Tf2 (blue circles). Tf2 causes a significant hyperpolarizing (∆ −6.3 mV) shift in the voltage dependence of activation and decreased the slope factor (∆ −1.3) (*n* = 11–14 cells). Data presented as mean ± SEM.

To examine the potency and selectivity of synthetic Tf2, we used a high-throughput fluorescence-based assay to measure changes in the membrane potential at hNa_V_1.1–1.7 expressed heterologously in HEK293 cells. Synthetic Tf2 potently activated hNa_V_1.3, with an EC_50_ of 213 ± 57 nM (Figure 1A). Consistent with the activity previously reported for venom-derived Tf2 [14], synthetic Tf2 retained selectivity for hNa_V_1.3, with no effect on hNa_V_1.1, hNa_V_1.2, or hNa_V_1.4–1.7 up to 10 µM (Figure 1A), making it more than 50-fold selective over these isoforms. To assess the mechanism of action of synthetic Tf2, we used whole-cell patch-clamp electrophysiology in HEK293 cells expressing hNa_V_1.3. Consistent with the β-scorpion activity previously reported for the native toxin, synthetic Tf2 (hereafter Tf2) at 1 µM caused a decrease in the peak current and a hyperpolarizing shift (Δ −11.3 mV) in the voltage dependence of activation (V_50_ control: −29.5 ± 0.7 mV; V_50_ Tf2: −40.8 ± 1.3 mV; *p* < 0.05; Figure 1B,C). The slope factor of the conductance–voltage curve also significantly increased in the presence of Tf2 at 1 µM, indicating a higher conductance at more hyperpolarizing membrane potentials (control: 3.6 ± 0.6 mV; Tf2: 8.0 ± 1.1 mV; *p* < 0.05; Figure 1C).

It has previously been shown that Tf2 does not alter the voltage dependence of activation of hNa_V_1.7 or hNa_V_1.8 expressed in *Xenopus* oocytes [14]. We thus sought to confirm this in mammalian cells and extend these studies to include the human Na_V_1.9 isoform. For this, we utilized the previously described hNav1.9_C4 construct in which the C-terminus from rNa_V_1.4 replaces the C-terminus of hNa_V_1.9, enabling trafficking of the channel to the cell surface [8,20]. Consistent with previous reports, 1 µM Tf2 does not shift the voltage dependence of activation at hNa_V_1.7 (V_50_ control: −23.1 ± 0.6 mV; V_50_ Tf2: −24.3 ± 0.7 mV; *p* > 0.05; Figure 1D) or hNa_V_1.8 (V_50_ control: −1.1 ± 0.3 mV; V_50_ Tf2: −1.2 ± 0.4 mV; *p* > 0.05; Figure 1E). Interestingly, 1 µM Tf2 was active at hNav1.9_C4, causing a hyperpolarizing shift (Δ −6.3 mV) in the voltage dependence of activation (V_50_ control: −55.1 ± 1.9 mV; V_50_ Tf2 −61.4 ± 1.2 mV; *p* < 0.05, Figure 1F) and a change (Δ −1.3) in the slope factor (control: 8.8 ± 0.4; Tf2: 7.6 ± 0.2; *p* < 0.05, Figure 1F). Given the novel activity at hNav1.9_C4, we also assessed whether Tf2 affects any other parameters of hNav1.9_C4 channel kinetics. Tf2 did not change the current density (control: 61.8 ± 12.6 pA/pF; Tf2: 68.1 ± 10.3 pA/pF; *p* > 0.05), or the voltage dependence of fast inactivation (V_50_ control: −98.16 ± 1.9 mV; V_50_ Tf2 −98.16 ± 1.5 mV; *p* > 0.05) or deactivation (see Figure S2A–C).

### 3.3. Tf2[S14R] has Reduced Excitatory Activity at Na_V_1.3

**Figure 2:**
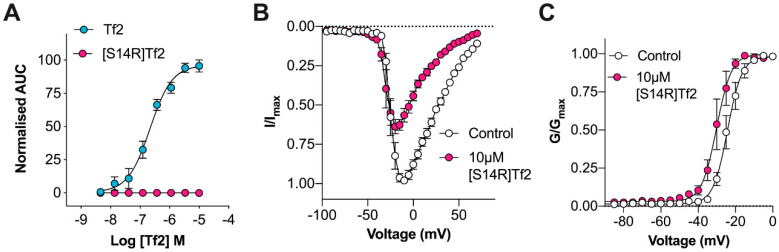
Tf2[S14R] loses excitatory activity at hNa_V_1.3. (**A**) Comparative activity of Tf2 and Tf2[S14R] on hNa_V_1.3 assessed using the FLIPR^Tetra^ membrane potential assay. Tf2 (blue circles) concentration-dependently increased the membrane potential (EC_50_ 213 ± 57 nM) while Tf2[S14R] (pink circles) had no effect up to 10 µM (*n* = 3–4 wells). (**B**) hNa_V_1.3 current–voltage relationship before (white circles) and after the addition of 10 μM Tf2[S14R] (pink circles). Tf2[S14R] decreased the peak current with a smaller effect on early channel opening compared to Tf2 (*n* = 5 cells). (**C**) hNa_V_1.3 conductance–voltage relationship before (white circles) and after the addition of 10 μM Tf2[S14R] (pink circles). Tf2[S14R] causes a smaller but significant (∆ −5.8 mV) hyperpolarizing shift in the voltage dependence of activation without affecting the slope factor (*n* = 5 cells). Data presented as mean ± SEM.

It has been demonstrated for several β-scorpion toxins that a single amino acid residue replacement of S14 (in Ts1) or E15 (in Cn2 and Css4) with an arginine can uncouple the ‘excitatory’ activity from the ‘inhibitory’ activity, producing β-scorpion toxin mutants that inhibit the peak current without causing early channel opening [11,20,21]. We therefore synthesized a Tf2[S14R] mutant and assessed activity at hNa_V_1.3. Unlike Tf2, the S14R mutant, at concentrations up to 10 µM, did not activate hNa_V_1.3 in a fluorescence-based membrane potential assay (Figure 2A). In patch-clamp electrophysiology, 10 µM Tf2[S14R] caused a reduction in peak current, although a small hyperpolarizing shift (Δ −5.8 mV) in the voltage dependence of activation remained (control: −24.3 ± 0.4 mV; Tf2[S14R]: −30.1 ± 0.7 mV; *p* < 0.05; Figure 2B,C). However, unlike wildtype Tf2, the slope factor of the conductance–voltage curve was not significantly different in the presence of Tf2[S14R] at 10 µM, indicating the S14R mutant had lost significant excitatory activity (control: 3.9 ± 0.4; Tf2[S14R]: 4.0 ± 0.6; *p* > 0.05; Figure 2C).

### 3.4. Tf2 Induces Calcium Influx in DRG Neurons

**Figure 3:**
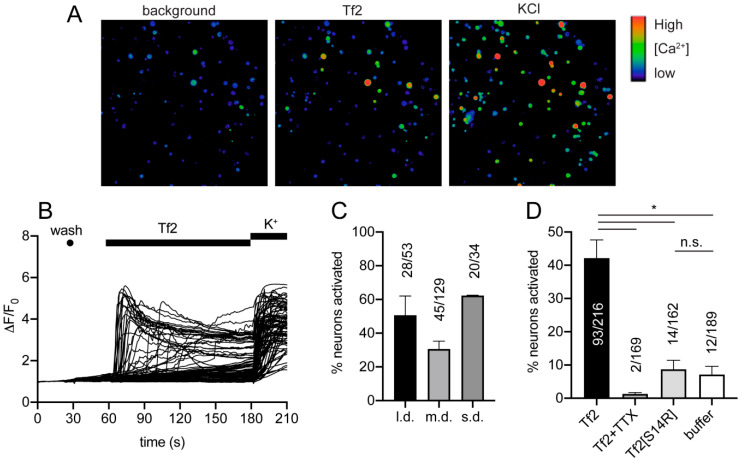
Tf2 activates mouse dorsal root ganglion neurons. (**A**) DRG cells before (background) and after the addition of 1 µM Tf2 and 30 mM KCl. (**B**) Traces from all neurons (defined as cells responding to 30 mM KCl and/or 1 µM Tf2) of one representative experiment. (**C**) Percentage of large (>600 µm^2^), medium (300–600 µm^2^), and small diameter (<300 µm^2^) neurons activated in the presence of 1 µM Tf2. (**D**) Percentage of neurons (total) activated in the presence of 1 µM Tf2, 1 µM Tf2 + 1 µM TTX, 1 µM Tf2[S14R], or buffer (negative control). Data are presented as SEM with the total number of neurons from 2–3 independent experiments; l.d., large diameter, m.d., medium diameter; s.d., small diameter; K^+^, 30 mM KCl; *, *p* < 0.05 using unpaired *t*-test; n.s., not significant.

It has been reported that Na_V_1.3 is expressed at low levels in the adult rodent peripheral nervous system [4]. Application of 1 µM Tf2 to isolated mouse DRG neurons caused an immediate, rapid, and sustained increase in the intracellular Ca^2+^ concentration ([Ca^2+^]*_i_*) in a subset of neurons (Figure 3A,B). Small, medium, and large diameter neurons were activated (Figure 3C). Activation was completely blocked (*p* < 0.05) in the presence of 1 µM TTX (Figure 3D), indicating that the effects of Tf2 on Ca^2+^ influx in mouse DRG neurons is dependent on the expression of TTX-sensitive VGSCs. At the same concentration (1 µM), Tf2[S14R] was not significantly different to cells treated with the negative control (*p* = 0.71), indicating that this analogue lacks the capacity to activate DRG neurons, consistent with its reduced excitatory activity of Na_V_1.3.

### 3.5. Tf2 Causes Spontaneous Pain and Swelling In Vivo

**Figure 4:**
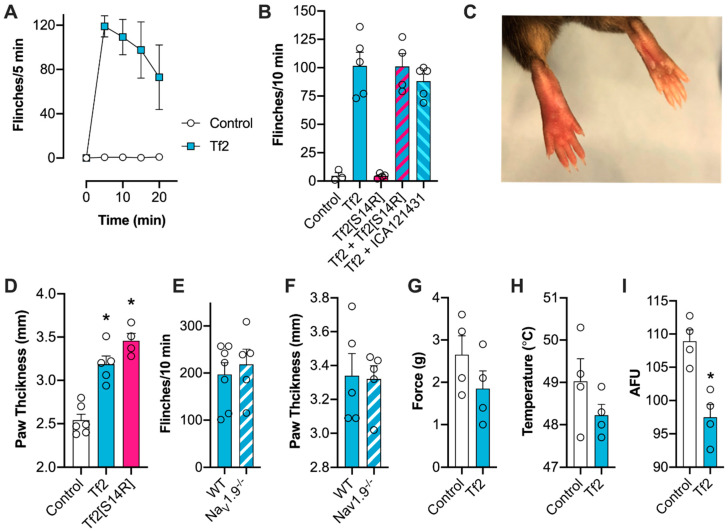
Intraplantar injection of Tf2 causes spontaneous pain behaviors and local swelling in mice (**A**) Time course of spontaneous pain behaviors induced by Tf2 (1 µM i.pl.). (**B**) Intraplantar injection of Tf2 (1 µM) causes spontaneous pain behaviors while intraplantar injection of Tf2[S14R] (10 µM) does not. Co-injection of Tf2[S14R] (10 µM) with Tf2 (1 µM) or ICA-121431 (500 nM) causes no reduction in spontaneous pain behaviors. (**C**) Intraplantar injection of Tf2 (left hind paw) causes local erythema (redness) and swelling. (**D**) Intraplantar injection of both Tf2 (1 µM) and Tf2[S14R] (10 µM) causes a significant increase in paw swelling. (**E**) Tf2-induced spontaneous pain behaviors are not attenuated in Na_V_1.9^-/-^ mice. (**F**) Tf2-induced swelling is not attenuated in Na_V_1.9^-/-^ mice. (**G**) Intraplantar injection of Tf2 (1 µM) has no effect on mechanical thresholds. (**H**) Intraplantar injection of Tf2 (1 µM) has no effect on heat thresholds. (**I**) Intraplantar injection of Tf2 (1 µM) causes a reduction in weight bearing (as measured by arbitrary fluorescence units; AFU). Data are presented as mean ± SEM with individual values plotted (*n* = 3–7 mice). Statistical significance was determined using *t*-test or one-way ANOVA with Dunnett’s post-test as appropriate, * *p* < 0.05 compared to control or WT.

Intraplantar injection of 1 µM (40 µL) Tf2 in mice caused a rapid induction of spontaneous pain behaviors, including flinching, licking, and shaking of the injected hind paw, that slowly subsided over 20 min (Figure 4A,B). In contrast, 10 µM Tf2[S14R] caused no spontaneous pain behaviors, consistent with the loss of excitatory activity at Na_V_1.3 and in DRG neurons seen in vitro (flinches/10 min: control, 5 ± 3; Tf2, 102 ± 12; Tf2[S14R], 5 ± 1; *p* < 0.05, Figure 4B). To assess if 10 µM (40 µL) Tf2[S14R] could compete with wild-type toxin for Na_V_1.3 binding in vivo, we co-administered it with 1 µM (40 µL) Tf2; however, it had no effect on Tf2-induced spontaneous flinching (flinches/10 min: Tf2 + Tf2[S14R] 101 ± 11; Figure 4B). In addition, the Na_V_1.1/1.3 inhibitor ICA-121431 (500 nM) had no effect on Tf2-induced spontaneous flinching (flinches/10 min: Tf2 + ICA-121431 88 ± 6; *p* > 0.05; Figure 4B), suggesting the flinching is not mediated via Na_V_1.3. Spontaneous pain was accompanied by erythema (redness) and swelling (Figure 4C), which occurred immediately after injection of Tf2 and also unexpectedly after Tf2[S14R], suggesting that this inflammatory response is not mediated via activation of Na_V_1.3 but instead through an additional target (paw thickness: control, 2.5 ± 0.2 mm; Tf2, 3.2 ± 0.1 mm; Tf2[S14R], 3.5 ± 0.1 mm; *p* < 0.05; Figure 4D). As Tf2 also activated hNa_V_1.9_C4, we assessed whether activation of Na_V_1.9 contributed to Tf2-induced spontaneous pain and swelling in vivo using Na_V_1.9^-/-^ mice. Compared to wildtype (WT) controls, Na_V_1.9^-/-^ mice did not exhibit a significant reduction in spontaneous pain behaviors (flinches/10 min: WT, 197 ± 25; Na_V_1.9^-/-^, 218 ± 32; *p* > 0.05) or paw swelling (paw thickness: WT, 3.3 ± 0.1 mm; Na_V_1.9^-/-^, 3.3 ± 0.1 mm; *p* > 0.05) after injection of Tf2 (Figure 4E,F). Once spontaneous pain subsided (~40 min post injection), we also assessed the effect of intraplantar Tf2 on mechanical thresholds, thermal thresholds, and weight bearing behavior in WT mice. Tf2 had no significant effect on mechanical thresholds (paw withdrawal threshold (PWT): control, 2.7 ± 0.4 g; Tf2, 1.9 ± 0.4 g; *p* > 0.05; Figure 4G) or paw thermal thresholds (PWT: control, 49.0 ± 0.5 °C; Tf2, 48.2 ± 0.3 °C; *p* > 0.05; Figure 4H) but did cause a significant reduction in weight bearing (arbitrary fluorescent unit (AFU): control, 108.9 ± 1.7; Tf2, 97.5 ± 1.9; *p* < 0.05; Figure 4I).

## 4. Discussion

Here, we describe for the first time the activity of synthetic β-scorpion toxin Tf2 on the full panel of human VGSCs (Na_V_1.1–1.9) constitutively expressed in mammalian cells, on the calcium influx in primary sensory DRG neurons, and on nociceptive behaviors in mice in vivo, with the overall aim to investigate its suitability as a selective Na_V_1.3 pharmacological probe. Modern chemical peptide synthesis strategies applied to α- and β-long chain scorpion toxins have allowed the production of these molecules on the multi-milligram scale and have allowed structural and functional studies that are difficult to perform with the small quantities usually isolated from scorpion venom [22,23,24,25]. Synthetic Tf2 faithfully reproduced the activity and mode-of-action of venom-derived Tf2 reported previously, strongly suggesting that both samples are also structurally identical. Whilst previously described as a selective Na_V_1.3 activator [14], we found that Tf2 had additional activity at hNa_V_1.9_C4, which is a TTX-resistant isoform preferentially expressed on small diameter DRG neurons that is associated with both ‘painless’ and ‘painful’ channelopathies [26,27].

The pharmacology of scorpion toxins at Na_V_1.9 remains largely unexplored, partly due to difficulties in the functional expression of Na_V_1.9 in heterologous systems. However, the β-scorpion toxin Ts1 (also known as TsVII), which has 73.4% sequence identity to Tf2, is also active on Na_V_1.9 [28]. This suggests that activity at Na_V_1.9 may be broadly conserved amongst other related β-scorpion toxins, but this remains to be assessed. Despite shifting the voltage dependence of activation at Na_V_1.9 (Δ −6.3 mV), Tf2-induced spontaneous pain and swelling developed normally in Na_V_1.9^-/-^ mice, indicating that this isoform had a minimal contribution to in vivo activity. This result was somewhat surprising, given the mutation L1158P in human Na_V_1.9 causes a similar hyperpolarizing shift in activation (Δ −6.7 mV), and has been linked to increased excitability of DRG neurons and painful peripheral neuropathy [29]. However, this mutation also slows deactivation, which was a channel parameter unaffected by Tf2, suggesting that changes in Na_V_1.9 deactivation may be important for neuronal excitability.

Selective VGSC modulators remain useful tool compounds for delineating the contribution of particular isoforms to the excitability of neurons in both physiological and pathological states [17,30]. Despite being implicated in neuropathic pain and epilepsy, there is a lack of highly potent and selective inhibitors of Na_V_1.3 available. The small molecule ICA-121431 is the most potent Na_V_1.3 inhibitor described to the date, but off-target activity at Na_V_1.1, which is also expressed on peripheral and central neurons, limits its use as a research tool in vivo [31]. In an attempt to rationally design a selective Na_V_1.3 inhibitor, we synthesized Tf2[S14R], where we mutated the serine at position 14 to a positively charged arginine. In the homologous β-scorpion toxin Ts1, mutation of S14 to an arginine resulted in a toxin that could still bind to Na_V_1.4 but no longer shifted the voltage dependence of activation [32]. In line with this observation, Tf2[S14R] inhibited the peak current of Na_V_1.3 without causing the same early channel opening as the native Tf2; however, it was unable to reverse Tf2-induced spontaneous pain behaviors in vivo. This is possibly due to the mutation causing a loss in potency, as the related toxin Cn2[E15R] required application at a concentration 200 times higher than the native to inhibit Na_V_1.6 in vivo [11]. Unfortunately, because the potency of Tf2 is already relatively weak, and Tf2[S14R] caused paw swelling at 10 µM, higher concentrations could not be assessed.

Tf2 caused Ca^2+^ influx in both small and large mouse adult DRG neurons, which was surprising given Na_V_1.3 is only expressed at significant levels in embryonic sensory neurons or in adult neurons after axotomy [4]. Interestingly, colonic neurons isolated from mouse thoracolumbar (T10-L1) and lumbosacral (L5-S2) DRGs express Na_V_1.3 mRNA transcripts at similar levels to Na_V_1.1 and Na_V_1.2 [33]. As DRG neurons in this study were isolated from all spinal levels, we cannot rule out the presence of these Na_V_1.3-expressing neurons in culture. However, given the definitive lack of proteomic evidence for Na_V_1.3 expression in adult uninjured DRG neurons, and that Tf2 activated a large proportion of all DRG neurons, it is more likely that the observed effect on Ca^2+^ influx is due to activity at other neuronal targets. This is consistent with Tf2-induced spontaneous flinching not being reversed by the Na_V_1.1/1.3 inhibitor ICA-121431 in vivo. Indeed, it is not uncommon for scorpion toxins to have promiscuous activity on both VGSCs and voltage-gated potassium (K_V_) channels, and blockade of a K_V_ channel could also result in Ca^2+^ influx [34,35]. Furthermore, there are scorpion toxins that modulate members of the family of voltage-gated calcium channels (Ca_V_), such as kurtoxin from *Parabuthus transvaalicus* [36]; however, the activity of Tf2 at voltage-gated potassium and calcium channels in heterologous expression systems remains to be tested. Inhibition of Tf2-induced Ca^2+^ influx by TTX could also be explained by a global depression of neuronal excitability rather than direct opposition of Tf2-induced Na_V_1.3 activation. The observation that Tf2[S14R], which lost activity at Na_V_1.3, also lost excitatory activity in DRG neurons suggests that the pharmacophore of Tf2 at this other neuronal target(s) overlaps at least partially with that of Na_V_1.3. In addition, the observation that Tf2[S14R], which lost excitatory activity in DRG neurons, still caused swelling in vivo suggests that Tf2-induced inflammation is mediated by an additional non-neuronal target. Scorpion venom- or toxin-induced inflammatory responses have been described previously and have been linked to the activation of innate immune cells via interaction with pattern recognition receptors [37]. The use of global and sensory neuron-specific Na_V_1.3 knockout mice would be required to tease out what contribution, if any, Na_V_1.3 has to Tf2-induced pain and inflammation.

In summary, Tf2 does not appear to be a suitable tool to study the effects of Nav1.3 channel activation in sensory neurons. This is due to additional activity at Na_V_1.9 and the probability of other neuronal and non-neuronal targets. Results from in vivo experiments that use Tf2 should be interpreted accordingly. There is, however, scope to use Tf2 in studying the activity of Na_V_1.9 in vitro, particularly in heterologous expression systems. Indeed, very few toxins to date are known to modulate the Na_V_1.9 isoform. Finally, the somewhat surprising finding that Tf2 and Tf2[S14R] cause inflammation in vivo could be explored further to understand how scorpion toxin envenomation affects inflammatory processes in humans.

## Supplementary Materials

Supplementary materials can be found at https://www.mdpi.com/2227-9059/8/6/155/s1. Figure S1: NMR secondary Hα chemical shift analysis of synthetic Tf2. Figure S2: Activity of Tf2 on other channel parameters of hNa_V_1.9_C4.

## References

[1] Shen H., Zhou Q., Pan X., Li Z., Wu J., Yan N. (2017). Structure of a eukaryotic voltage-gated sodium channel at near-atomic resolution. Science.

[2] Catterall W.A., Goldin A.L., Waxman S.G. (2005). International Union of Pharmacology. XLVII. Nomenclature and structure-function relationships of voltage-gated sodium channels. Pharm. Rev..

[3] Cummins T.R., Aglieco F., Renganathan M., Herzog R.I., Dib-Hajj S.D., Waxman S.G. (2001). Nav1.3 sodium channels: Rapid repriming and slow closed-state inactivation display quantitative differences after expression in a mammalian cell line and in spinal sensory neurons. J. Neurosci..

[4] Waxman S.G., Kocsis J.D., Black J.A. (1994). Type III sodium channel mRNA is expressed in embryonic but not adult spinal sensory neurons, and is reexpressed following axotomy. J. Neurophysiol..

[5] Craner M.J., Klein J.P., Renganathan M., Black J.A., Waxman S.G. (2002). Changes of sodium channel expression in experimental painful diabetic neuropathy. Ann. Neurol..

[6] Black J.A., Cummins T.R., Plumpton C., Chen Y.H., Hormuzdiar W., Clare J.J., Waxman S.G. (1999). Upregulation of a silent sodium channel after peripheral, but not central, nerve injury in DRG neurons. J. Neurophysiol..

[7] Samad O.A., Tan A.M., Cheng X., Foster E., Dib-Hajj S.D., Waxman S.G. (2013). Virus-mediated shRNA knockdown of Na(v)1.3 in rat dorsal root ganglion attenuates nerve injury-induced neuropathic pain. Mol. Ther..

[8] Goral R.O., Leipold E., Nematian-Ardestani E., Heinemann S.H. (2015). Heterologous expression of Na1.9 chimeras in various cell systems. Pflug. Arch..

[9] Minett M.S., Falk S., Santana-Varela S., Bogdanov Y.D., Nassar M.A., Heegaard A.M., Wood J.N. (2014). Pain without nociceptors? Nav1.7-independent pain mechanisms. Cell Rep..

[10] Nassar M.A., Baker M.D., Levato A., Ingram R., Mallucci G., McMahon S.B., Wood J.N. (2006). Nerve injury induces robust allodynia and ectopic discharges in Nav1.3 null mutant mice. Mol. Pain.

[11] Israel M.R., Morgan M., Tay B., Deuis J.R. (2018). Toxins as tools: Fingerprinting neuronal pharmacology. Neurosci. Lett..

[12] Bosmans F., Tytgat J. (2007). Voltage-gated sodium channel modulation by scorpion alpha-toxins. Toxicon.

[13] Pedraza Escalona M., Possani L.D. (2013). Scorpion beta-toxins and voltage-gated sodium channels: Interactions and effects. Front. Biosci. (Landmark Ed.).

[14] Camargos T.S., Bosmans F., Rego S.C., Mourao C.B., Schwartz E.F. (2015). The Scorpion Toxin Tf2 from Tityus fasciolatus Promotes Nav1.3 Opening. PLoS ONE.

[15] Cestele S., Qu Y., Rogers J.C., Rochat H., Scheuer T., Catterall W.A. (1998). Voltage sensor-trapping: Enhanced activation of sodium channels by beta-scorpion toxin bound to the S3-S4 loop in domain II. Neuron.

[16] Robinson S.D., Mueller A., Clayton D., Starobova H., Hamilton B.R., Payne R.J., Vetter I., King G.F., Undheim E.A.B. (2018). A comprehensive portrait of the venom of the giant red bull ant, Myrmecia gulosa, reveals a hyperdiverse hymenopteran toxin gene family. Sci. Adv..

[17] Israel M.R., Tanaka B.S., Castro J., Thongyoo P., Robinson S.D., Zhao P., Deuis J.R., Craik D.J., Durek T., Brierley S.M. (2019). NaV 1.6 regulates excitability of mechanosensitive sensory neurons. J. Physiol..

[18] Deuis J.R., Vetter I. (2016). The thermal probe test: A novel behavioral assay to quantify thermal paw withdrawal thresholds in mice. Temperature.

[19] Yin K., Deuis J.R., Lewis R.J., Vetter I. (2016). Transcriptomic and behavioural characterisation of a mouse model of burn pain identifies the cholecystokinin 2 receptor as an analgesic target. Mol. Pain.

[20] Sizova D.V., Huang J., Akin E.J., Estacion M., Gomis-Perez C., Waxman S.G., Dib-Hajj S.D. (2020). A 49-residue sequence motif in the C terminus of Nav1.9 regulates trafficking of the channel to the plasma membrane. J. Biol. Chem..

[21] Karbat I., Ilan N., Zhang J.Z., Cohen L., Kahn R., Benveniste M., Scheuer T., Catterall W.A., Gordon D., Gurevitz M. (2010). Partial agonist and antagonist activities of a mutant scorpion beta-toxin on sodium channels. J. Biol. Chem..

[22] Kubota T., Durek T., Dang B., Finol-Urdaneta R.K., Craik D.J., Kent S.B., French R.J., Bezanilla F., Correa A.M. (2017). Mapping of voltage sensor positions in resting and inactivated mammalian sodium channels by LRET. Proc. Natl. Acad. Sci. USA.

[23] Dang B., Kubota T., Mandal K., Correa A.M., Bezanilla F., Kent S.B. (2016). Elucidation of the Covalent and Tertiary Structures of Biologically Active Ts3 Toxin. Angew. Chem. Int. Ed. Engl..

[24] Durek T., Vetter I., Wang C.I., Motin L., Knapp O., Adams D.J., Lewis R.J., Alewood P.F. (2013). Chemical engineering and structural and pharmacological characterization of the alpha-scorpion toxin OD1. ACS Chem. Biol..

[25] Israel M.R., Thongyoo P., Deuis J.R., Craik D.J., Vetter I., Durek T. (2018). The E15R Point Mutation in Scorpion Toxin Cn2 Uncouples Its Depressant and Excitatory Activities on Human NaV1.6. J. Med. Chem..

[26] Dib-Hajj S.D., Tyrrell L., Black J.A., Waxman S.G. (1998). NaN, a novel voltage-gated Na channel, is expressed preferentially in peripheral sensory neurons and down-regulated after axotomy. Proc. Natl. Acad. Sci. USA.

[27] Dib-Hajj S.D., Black J.A., Waxman S.G. (2015). NaV1.9: A sodium channel linked to human pain. Nat. Rev. Neurosci..

[28] Bosmans F., Puopolo M., Martin-Eauclaire M.F., Bean B.P., Swartz K.J. (2011). Functional properties and toxin pharmacology of a dorsal root ganglion sodium channel viewed through its voltage sensors. J. Gen. Physiol..

[29] Huang J., Han C., Estacion M., Vasylyev D., Hoeijmakers J.G., Gerrits M.M., Tyrrell L., Lauria G., Faber C.G., Dib-Hajj S.D. (2014). Gain-of-function mutations in sodium channel Na(v)1.9 in painful neuropathy. Brain J. Neurol..

[30] Deuis J.R., Dekan Z., Wingerd J.S., Smith J.J., Munasinghe N.R., Bhola R.F., Imlach W.L., Herzig V., Armstrong D.A., Rosengren K.J. (2017). Pharmacological characterisation of the highly NaV1.7 selective spider venom peptide Pn3a. Sci. Rep..

[31] McCormack K., Santos S., Chapman M.L., Krafte D.S., Marron B.E., West C.W., Krambis M.J., Antonio B.M., Zellmer S.G., Printzenhoff D. (2013). Voltage sensor interaction site for selective small molecule inhibitors of voltage-gated sodium channels. Proc. Natl. Acad. Sci. USA.

[32] Kubota T., Dang B., Carvalho-de-Souza J.L., Correa A.M., Bezanilla F. (2017). Nav channel binder containing a specific conjugation-site based on a low toxicity beta-scorpion toxin. Sci. Rep..

[33] Hockley J.R., Gonzalez-Cano R., McMurray S., Tejada-Giraldez M.A., McGuire C., Torres A., Wilbrey A.L., Cibert-Goton V., Nieto F.R., Pitcher T. (2017). Visceral and somatic pain modalities reveal NaV 1.7-independent visceral nociceptive pathways. J. Physiol..

[34] Liu P., Jo S., Bean B.P. (2012). Modulation of neuronal sodium channels by the sea anemone peptide BDS-I. J. Neurophysiol..

[35] Klint J.K., Senff S., Rupasinghe D.B., Er S.Y., Herzig V., Nicholson G.M., King G.F. (2012). Spider-venom peptides that target voltage-gated sodium channels: Pharmacological tools and potential therapeutic leads. Toxicon.

[36] Sidach S.S., Mintz I.M. (2002). Kurtoxin, a gating modifier of neuronal high- and low-threshold ca channels. J. Neurosci..

[37] Reis M.B., Zoccal K.F., Gardinassi L.G., Faccioli L.H. (2019). Scorpion envenomation and inflammation: Beyond neurotoxic effects. Toxicon.

