# Supplementary material for: Characterization of Synthetic Tf2 as a NaV1.3 Selective Pharmacological Probe
Source: Biomedicines. 2020 Jun 11;8(6):155. doi: 10.3390/biomedicines8060155 (PMC7345637; doi:10.3390/biomedicines8060155)
Supplement: Supplementary file 1 [file biomedicines-08-00155-s001.pdf]

Supplementary material:

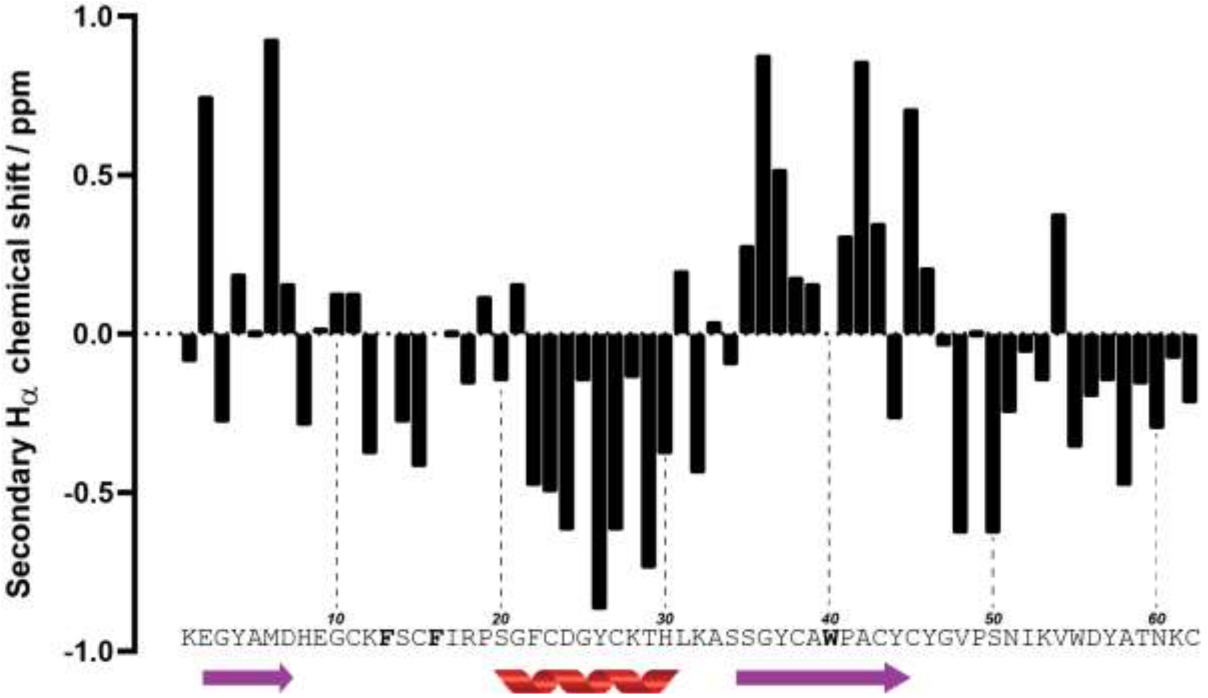

Figure S1: NMR secondary Hα chemical shift analysis of synthetic Tf2. Chemical shifts of the H proton of each residue (except those highlighted in bold) were determined by sequential assignment of 2D-NOESY and TOCSY spectra and compared to random coil values [1]. Positive secondary H chemical shifts indicate -strand structure, whereas negative values indicate -helical structure.

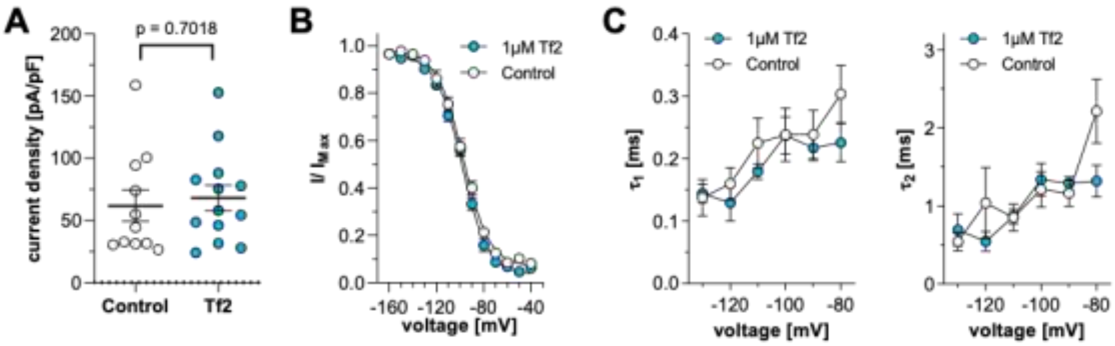

Figure S2: Activity of Tf2 on other channel parameters of hNav1.9_C4. **(A)** Effect of Tf2 (1 μM) (blue circles) on current density, compared to buffer control (0.1% BSA) (white circles). **(B)** Effect of Tf2 (1 μM) (blue circles) on voltage-dependence of fast inactivation (n = 8-12), compared to buffer control (0.1% BSA) (white circles) **(C)** Effect of Tf2 (1 μM) (blue circles) on the current decay of deactivation (n = 6-12), compared to buffer control (0.1% BSA) (white circles). Data are presented as mean ± SEM.

References:

1. Wishart, D.S.; Bigam, C.G.; Holm, A.; Hodges, R.S.; Sykes, B.D. 1H, 13C and 15N random coil NMR chemical shifts of the common amino acids. I. Investigations of nearest-neighbor effects. *Journal of biomolecular NMR* **1995**, *5*, 67-81, doi:10.1007/bf00227471.
